# Protocol for “Genetic composition of sickle cell disease in the Arab population: A systematic review”

**DOI:** 10.1002/hsr2.450

**Published:** 2022-05-03

**Authors:** Fateen Ata, Zohaib Yousaf, Sundus Sardar, Saad Javed, Phool Iqbal, Ibraheem Khamees, Lujain Salahaldeen Malkawi, Mohamed A. Yassin

**Affiliations:** ^1^ Department of Internal Medicine Hamad General Hospital, Hamad Medical Corporation Doha Qatar; ^2^ Department of Internal Medicine Allama Iqbal Medical College Lahore Pakistan; ^3^ Department of Internal Medicine, Faculty of Medicine Jordan University of Science and Technology Irbid Jordan; ^4^ Department of Medical Oncology/Hematology National Centre for Cancer Care and Research, Hamad Medical Corporation Doha Qatar

**Keywords:** Arab, genetics, genotype, middle east, SCD, sickle cell disease

## Abstract

**Background:**

Sickle Cell Disease (SCD) is a global health issue in hematology with a progressively increasing prevalence. There are recent advances in the management of SCD, with new drugs being introduced. It is essential to analyze the genetic makeup of SCD regionally to anticipate the effectiveness of management modalities. This systematic review's main objectives are (a) to combine the existing knowledge of the genetic composition of SCD in the Arab population and (b) to analyze the various phenotypes of SCD prevalent in the Arab population.

**Methods:**

We will perform a systematic review and search multiple electronic databases predefined search terms to identify eligible articles. Eligible studies should report findings on the genetic testing of Sickle Cell disease in the 22 Arab countries. Case reports, case series, observational studies with cross‐sectional or prospective research design, case‐control studies, and experimental studies will be included. Study quality will be independently evaluated by two reviewers using the statistical methodology and categories guided by the Cochrane Collaboration Handbook and PRISMA guidelines.

**Discussion:**

This review will explore and integrate the evidence available on the various genotypes and phenotypes of SCD in the Arab population. By acquiring and summarizing data about the genetic and phenotypic variants of the SCD patient population, this study will add to the knowledge and help find more precise treatments.

**Systematic review registration:**

The protocol is registered at the International Prospective Register of Systematic Reviews (PROSPERO; registration number: CRD42020218666).

**Abbreviations**
GRADE: grading of recommendations assessment, development and evaluation
MOOSE: meta‐analyses of observational studies in epidemiology
PRISMA: preferred reporting items for systematic reviews and meta‐analyses
PROSPERO: international prospective register of systematic reviews
RCT: randomized controlled trial
SCD: sickle cell disease
SCT: stem cell transplant

## 1 INTRODUCTION

SCD is a spectrum of hereditary hemoglobinopathies characterized by abnormal hemoglobin S (HbS) polymer. This results in a cascade of sickling and unsickling erythrocytes, ultimately leading to hemolysis.^1^ Other than chronic hemolytic anemia, it can also present with acute episodes of crisis, the vaso‐occlusive crisis being the most common. The disease also carries a mortality burden secondary to life‐threatening complications such as acute chest syndrome.^2^ SCD is relatively prevalent in the Middle East, with some countries having the highest prevalence globally (2.1%).^3^ It is estimated that the global burden of SCD will increase by up to 30% by 2050.^4^

On the other hand, there is a recent advancement in the treatment of SCD. Some of the newly approved disease‐modifying agents include Voxelotor (1500 mg daily) and Crizanlizumab (5 mg/kg).^5^, ^6^ Additionally, there are recent trials on gene therapy in SCD with promising effectiveness.^7^ To analyze and anticipate treatment responsiveness of current therapies and to guide further studies focusing on gene therapy, it is vital to have an updated genetic database of SCD in the areas where it is prevalent. The last updated review on genotypes of SCD in the Arab population was published in 2011.^8^ Many studies describing genotypic and phenotypic variants of SCD have been published thereafter, creating a need to pool the updated genetic database of SCD in the 22 Arab countries. We will conduct a systematic review on all patients with reported genotypes of SCD from the Arab countries. Our primary outcome will be the genotypes, and secondary outcomes will be the mutational variations, haplotypes, and their correlations to phenotypes and treatments of SCD.

## 2 METHODS

This protocol is in accordance with the Preferred Reporting Items for Systematic Reviews and Meta‐Analyses (PRISMA) and PRISMA guidelines for Protocols (PRISMA‐P).^9^, ^10^ The protocol has been registered at the International Prospective Register of Systematic Reviews (PROSPERO; registration number: CRD42020218666).

## 3 DATA SOURCES, SEARCH TERMS AND STRATEGY, AND STUDY SELECTION

For achieving the study objectives, searches will be carried out in the following electronic databases: PubMed, Scopus, and Google Scholar. The following search terms will be used in the literature review: “Genotype” OR “Genetics” OR “Gene” OR “Mutations” OR “Haplotype” AND “Sickle Cell Disease” OR “SCD” OR “Hemoglobin S/O” OR “Sickle cell anemia” OR “SCA” OR “sickle/ beta‐thalassemia,” “SC/SD” OR “Hb SS” OR “Hb SC” OR “Hb Sβ+” OR “Hb Sβ0” OR “HbSD” OR “HbSE” OR “HbSO Arab” OR “HBS oman” AND “Arab” OR “Arab countries” OR “Arab Population” OR “The Middle East” OR “Algeria” OR “Bahrain” Comoros” OR “Djibouti” OR “Egypt” OR “Iraq” OR “Jordan” OR “Kuwait” OR “Lebanon” OR “Libya” OR “Mauritania” OR “Morocco” OR “Oman” OR “Palestine” OR “Qatar” OR “Saudi Arabia” OR “Somalia” OR “Sudan” OR “Syria” OR “Tunisia” OR “the United Arab Emirates” OR “Yemen.” Relevant articles will then be exported to endnote for screening based on inclusion and exclusion criteria and removal of duplicate citations. Four reviewers in two groups (F.A. and S.J., Z.Y., and S.S.) will screen the articles, and each group will cross‐check the screening of the other group. Any disagreements will be sorted out by a separate member (PI). The systematic method specifies that all published research constitutes the literature search on genotypes of SCD. The search strategy is considered adequate to minimize the risk of selection and detection bias. After the initial screening by title, abstract, and keywords, the same group of reviewers will screen the full‐text articles. Additionally, relevant articles will be added from the manual screening of the references if they fulfill the inclusion criteria.

A populated PRISMA‐P checklist was used as an aid to authors to clearly, completely, and transparently let reviewers and readers know what authors intend to do.^10^

## 4 INCLUSION AND EXCLUSION CRITERIA

### 4.1 Types of studies

Eligible studies should report the genetic variants of SCD in the Arab population. All literature, including clinical trials, case reports, case series, and observational studies (retrospective and prospective) from any date till November 2020 in the English language, will be included. Studies in languages other than English will not be included.

### 4.2 Participants

The study population will include pediatric (≤18 years), as well as adult (>18 years) patients diagnosed with SCD and having genetic testing results.

Exclusion criteria will include patients with SCD who do not have genetic testing done or results not reported.

## 5 DATA EXTRACTION

Data from the finalized articles will be extracted into a data extraction excel sheet by six reviewers (F.A., P.I., S.J., L.M., I.K., S.S., and Z.Y.). The data collection sheet will include digital object identifier, author and year of publication, number of patients, patient demographics such as age, gender, body mass index. It will also include hemoglobin electrophoresis results, genotypes, phenotypes, and treatment of SCD. We will also include the type of encounter, that is, inpatient or outpatient, where available. All the reported genotypes of SCD will be included, such as Hb SS, Hb SC, Hb Sβ+, Hb Sβ0, HbSD, HbSE, HbSO, and other rare types. We will also collect all described mutations in these patients. Phenotypic data will include hemolytic anemia, pain crisis, acute splenic sequestration, functional asplenia, hyperviscosity, transfusional hemosiderosis, neurological, ophthalmologic, pulmonary, renal, hepatobiliary, musculoskeletal, and dermatological manifestations. Treatments will include exchange transfusion, deferasirox, SCT, hydroxyurea, L‐Glutamine, Voxelotor, Crizanlizumab, and Gene therapy.

Collected data by each reviewer will be double‐checked by another reviewer from the group in order to increase the accuracy of data reporting. Descriptive statistics will be used to describe the socio‐demographic and clinical parameters of the population, with continuous variables presented as means (±SD) or Median (interquartile range) as appropriate. All data will be entered in duplicate and cross‐checked for accuracy, and disparities will be discussed in a team meeting to minimize data entry errors. Assessment of study quality or strength of study will be carried out (high, moderate, low).

## 6 ASSESSMENT OF METHODOLOGICAL QUALITY (RISK OF BIAS)

Study quality will be evaluated by two reviewers (F.A. and M.A.Y.) using the statistical methodology and categories described in the Cochrane Collaboration Handbook, PRISMA, and other applicable guidelines. In case of disagreement, a group meeting will be arranged to reach a conclusion. Potential issues such as baseline imbalance will also be evaluated. Various bias assessment tools will be used based on the type of studies, such as RoB 2.0: a revised tool to assess the risk of bias in randomized trials, and Grading of Recommendations Assessment, Development and Evaluation (GRADE).^11^, ^12^

## 7 DISCUSSION

Understanding of the genetic makeup of SCD has led to new insights into the pathophysiology, clinical course, and management of this severe hereditary anemia. Sickle cell disease is a common hemoglobinopathy in the Middle‐east and Africa. It has a spectrum of manifestations and complications involving almost every organ system.^13^ Treatment of SCD can majorly be divided into preventive and curative therapies. Hydroxyurea is the mainstay in the prevention of SCD complications and manifestations. It has a well‐established role in reducing vaso‐occlusive and pain crises, decreasing transfusions, and priapism frequencies. It is also associated with prolonged survival.^14^ Transfusion itself can lead to complications due to iron overload in SCD patients, which is why finding a curative treatment is an area of interest.^15^, ^16^ Preventive treatment focuses on minimizing the occurrence of complications such as vaso‐occlusive crisis and hemolysis. Historically stem cell transplant (SCT) has been the only curative treatment of SCD.^17^ There are currently at least 30 clinical trials, either ongoing or recently completed, focused on finding new drugs with various mechanisms of action to manage SCD and its complications.^18^ Additionally, researchers are focusing on developing genetic therapy as an alternative to SCT for curative management. Some of the therapies with particular focus include β‐globin gene addition (ClinicalTrials.gov Identifier: NCT02247843), γ‐globin gene addition, Targeted γ‐globin induction, and γ‐globin repressor silencing.^17^, ^19^

Success in finding a curative gene therapy relies heavily on understanding the genotypes of SCD to the deepest levels. The basis of genetic alteration in SCD lies in a homozygous missense mutation in the β‐globin gene resulting in the polymerization of hemoglobin S molecule.^20^ The main genotypes of SCD include sickle cell anemia, HbSC disease, S‐β^0^ thalassemia, S‐β^+^ thalassemia, HbSE disease, and sickle cell anemia‐α thalassemia.^21^ El‐Hazmi et al described the genotypes and phenotypes of SCD in the Arab population in 2011.^8^ Since then, there have been many new studies describing variants of SCD genotypes and phenotypes, and with the emerging era of gene therapy for SCD, it is essential to compile the current evidence, especially in the last 5 years.^22^, ^23^, ^24^, ^25^, ^26^, ^27^, ^28^, ^29^, ^30^, ^31^, ^32^, ^33^, ^34^, ^35^, ^36^, ^37^, ^38^, ^39^, ^40^, ^41^, ^42^, ^43^, ^44^ We will pool all data describing the genotypic and phenotypic variants of SCD in the Arab population to date in a systematic review. This will increase the understanding of the SCD in the Middle East, focusing on the Arab ethnicity, as well as provide a guide for gene therapy in the region.

### 7.1 Limitations

Methodological biases in the primary studies included may cause uncertainty in the results of the present study. We intend to collect data only from the Arab population; however, this will not be entirely representative of the global data as SCD is common in the African region also. This will be a descriptive study and hence will not include any data on clinical correlations of various variables in the patients with SCD.

## FUNDING

This study was not funded.

## CONFLICT OF INTEREST

The authors declare that they have no competing interests.

## AUTHORS' CONTRIBUTIONS

Conceptualization: Mohamed Yassin.

Data Curation: Fateen Ata.

Formal Analysis: Zohaib Yousaf.

Investigation: Fateen Ata, Zohaib Yousaf, Sundus Sardar, Saad Javed, Phool Iqbal, Ibrahim Khamees, Lujain Malkawi.

Methodology: Fateen Ata, Mohamed Yassin.

Project Administration: Fateen Ata, Mohamed Yassin.

Supervision: Mohamed Yassin.

Writing – Original Draft Preparation: Fateen Ata, Zohaib Yousaf, Sundus Sardar, Saad Javed, Phool Iqbal, Ibrahim Khamees, Lujain Malkawi, Mohamed Yassin.

Writing – Review & Editing: Fateen Ata, Mohamed Yassin.

All authors read and approved the final manuscript.

## ETHICS STATEMENT

Ethical approval is not required for this systematic review and meta‐analysis as only a secondary analysis of data already available in scientific databases will be conducted. The results of this review will be submitted for peer‐reviewed publication and presented at relevant conferences. Private information from individuals will not be published.

## Data Availability

Data sharing not applicable.
